# *Chromochloris zofingiensis* (Chlorophyceae) Divides by Consecutive Multiple Fission Cell-Cycle under Batch and Continuous Cultivation

**DOI:** 10.3390/biology10020157

**Published:** 2021-02-16

**Authors:** Idan Koren, Sammy Boussiba, Inna Khozin-Goldberg, Aliza Zarka

**Affiliations:** Microalgal Biotechnology Laboratory, French Associates Institute for Agriculture and Biotechnology of Drylands, The Jacob Blaustein Institutes for Desert Research, Ben-Gurion University of the Negev, Sede Boker Campus, Midreshet Ben-Gurion 8499000, Israel; idankorenx@gmail.com (I.K.); sammy@bgu.ac.il (S.B.); khozin@bgu.ac.il (I.K.-G.)

**Keywords:** astaxanthin, cell-cycle, *Chromochloris zofingiensis*, microalgae, multiple fission

## Abstract

**Simple Summary:**

Microalgae are plant-like micro-organisms naturally found in fresh and marine water environments, inhabiting a vast range of ecosystems. They capture light energy through photosynthesis and convert low energy inorganic compounds (carbon dioxide and water) into high energy complex organic compounds, such as carbohydrates and fats. *Chromochloris zofingiensis* is a unicellular microalga currently under intensive research, due to its ability to produce high value pharmaceutical and nutritional pigments. Understanding its growth characteristics is crucial for the establishment of an efficient commercial production of those pigments from this alga. Thus, we have developed a method to stain the nucleus of the alga which enabled us to follow the division pattern under commonly used cultivation methods. We found that *C. zofingiensis* cells conduct consecutive DNA synthesis and divisions of the nucleus to produce 8 or 16 nuclei before it divides into 8 or 16 daughter cells, respectively. Under high light illumination, the whole process lasts several days, through which cells grow during the light period and divide during the dark period. These findings can be assimilated for the development of the biotechnology process for high pigment productivity.

**Abstract:**

Several green algae can divide by multiple fission and spontaneously synchronize their cell cycle with the available light regime. The yields that can be obtained from a microalgal culture are directly affected by cell cycle events. *Chromochloris zofingiensis* is considered as one of the most promising microalgae for biotechnological applications due to its fast growth and the flexible trophic capabilities. It is intensively investigated in the context of bio-commodities production (carotenoids, storage lipids); however, the pattern of cell-cycle events under common cultivation strategies was not yet characterized for *C. zofingiensis*. In this study, we have employed fluorescence microscopy to characterize the basic cell-cycle dynamics under batch and continuous modes of phototrophic *C. zofingiensis* cultivation. Staining with SYBR green—applied in DMSO solution—enabled, for the first time, the clear and simple visualization of polynuclear stages in this microalga. Accordingly, we concluded that *C. zofingiensis* divides by a consecutive pattern of multiple fission, whereby it spontaneously synchronizes growth and cell division according to the available illumination regime. In high-light continuous culture or low-light batch culture, *C. zofingiensis* cell-cycle was completed within several light-dark (L/D) cycles (14 h/10 h); however, cell divisions were synchronized with the dark periods only in the high-light continuous culture. In both modes of cultivation, daughter cell release was mainly facilitated by division of 8 and 16-polynuclear cells. The results of this study are of both fundamental and applied science significance and are also important for the development of an efficient nuclear transformation system for *C. zofingiensis.*

## 1. Introduction

In addition to their recognized biotechnological potential [1,2,3], microalgae represent a unique group of unicellular organisms for the investigation of cell-cycle patterns. Some microalgae divide through binary fission, in which one cell divides into two cells, while others divide through multiple fission, in which a cell can produce up to several thousand daughter cells [4], however, in some cases, both binary and multiple fission can be executed in response to changes in ambient conditions or life cycle phase (reviewed by Bišová and Zachleder, 2014) [4].

The cell-cycle can be described as a sequence of events reoccurring in each cell leading to the production of daughter cells [5,6,7]. This perception has been offered as a better way to understand the multiple fission cell-cycle [4]. Mitchison [8] suggested that the cell-cycle can be presented as a sequence composed of two coordinated processes. The first is the G1 phase in which the cell grows in size, and the second is the DNA replication–division sequence (DNArds), in which the cells undergo S, G2, M, and C phases. The S, G2, M, and C phases represent DNA synthesis for replication, second growth phase, nuclear division through meiosis and daughter cell formation through cytokinesis, respectively. It is important to note that several G1 phases and DNArds can be conducted and can partly overlap during one cell-cycle. After approximately doubling in cell size (during the G1 phase), a commitment point (CP, commitment to divide) is attained and thereafter DNArds can be executed in the dark [4]. The mechanism/s by which the doubling in size is verified is not clear yet, though three models were proposed (for more details see [9]).

Two distinct patterns of multiple fission cell-cycle have been described [4]. The first is the consecutive pattern, characteristic to *Scenedesmus* and *Desmodesmus*, under which DNA replication and nuclear division are executed shortly after the attainment of CP; if several CPs were attained during one cell-cycle then several sessions of DNArds would occur in a consecutive pattern. Hence, the cell-cycle of microalgae expressing the consecutive pattern of multiple fission would include polynuclear cells (PNC). The second pattern is the clustered pattern of multiple fission cell-cycle typical to *Chlamydomonas reinhardtii*. In this type of multiple fission, nuclear division occurs soon after DNA replication and is immediately followed by cell division; consequently, it does not include polynuclear stages [10].

In microalgae capable of multiple fission, when conditions allow cells to grow and double in size, a number of CPs can be attained during one cell-cycle that will terminate in an equal number of DNArds [4]. Therefore, the number of daughter cells will be equal to 2 in the order of the number of CPs attained. In theory, this type of cell-cycle allows the illuminated period to be dedicated to photo-assimilation, anabolic processes, and growth, while cell divisions and the underlying processes facilitating them are postponed to the dark period, not to interfere with growth, and are protected from the harmful effects of solar radiation. Therefore, the multiple fission cell-cycle tends to spontaneously synchronize the growth (G1) and the division processes (DNArds) with the available illumination regime [4,11].

The multiple-fission cell-cycle is the main reproduction strategy of many green algae, including the genera *Desmodesmus*, *Chlorella*, *Chlamydomonas*, *Scenedesmus* and *Chromochloris*. Environmentally responsible and profitable microalgal biotechnology would utilize solar irradiance, therefore, a worthwhile attempt to produce biocommodities from microalgae would inevitably include a light/dark synchronized population that divides through one of the above-mentioned patterns of multiple fission. As cell-cycle events directly affect major culture parameters, including cell size, cell density and amount of storage compounds [4,11,12], cell-cycle characterization is of central importance for microalgae-based biotechnology.

*Chromochloris zofingiensis* ((Dönz) Fučíková K. et Lewis L.A.) stands out among the biotechnologically-interesting microalgae, as an alternative microalgal source of the high value pigment astaxanthin, since it can grow to high biomass densities through phototrophic, heterotrophic, or mixotrophic cultivation modes [13]. In addition, *C. zofingiensis* can tolerate media based on low-quality water and produce valuable biocommodities, such as lipids, starch, and the carotenoid pigments lutein, canthaxanthin, and astaxanthin [14,15,16,17,18]. Recently, the chromosome-level assembly of the genome [19] and time-resolved carotenoid profiling and transcriptomic analysis for *C. zofingiensis* were published [20,21]. These analyses advance the understanding of carotenoid production in this alga and enhance prospects for improving commercial production of *C. zofingiensis*.

Since it was isolated, *C. zofingiensis* was mostly investigated in the context of biotechnological applications associated with the production of pigments and oil, and only few investigations referred to cell-cycle-associated morphology. Fučíková and Lewis (2012) [22] resurrected the genus *Chromochloris,* previously affiliated with the genus *Chlorella*, by considering both morphological observations and phylogenetic analyses. Although several morphological and life history investigations were performed [19,22,23,24,25], the basic characteristics of *C. zofingiensis* reproduction under common cultivation techniques were not elucidated yet. Evidently, Fučíková and Lewis (2012) [22], despite performing a thorough literature review and direct attempts to stain nuclei, were unable to determine with complete certainty whether mature cells of the genus members contain several nuclei. It is only recently that Cryo-soft X-ray tomography visualized the nuclei in a cluster of cells, indicating that *C. zofingiensis* divides by multiple fission [19]. The same study also suggested a temporal separation between growth and division and indicated a similarity between *C. zofingiensis* and *C. reinhardtii* patterns of reproduction. The similarity, however, is not straightforward, as *Chlamydomonas* is affiliated with a different order within *Chlorophyceae*, *Chlamydomonadales*, whereas *C. zofingiensis* belongs to *Sphaeropleales* [4,19]. In addition, *C. reinhardtii* cell-cycle was characterized as the clustered multiple fission type [4]: under this type, no multinuclear stages are observed during the cell-cycle. *C. zofingiensis* cultures, however, include a variety of multinuclear stages, as will be shown in the results section. Therefore, additional investigation is needed to clarify *C. zofingiensis* cell-cycle pattern.

In this study, we set out to characterize the division pattern that is executed by *C. zofingiensis* in cultures grown phototrophically, both under batch and continuous cultivation modes and different illumination intensities. The methodology used in this study demonstrates an accessible and cost-efficient way to investigate the microalgal pattern of reproduction and showed that *C. zofingiensis* executes the consecutive cell-cycle division pattern which under the tested conditions is completed within several 24 h L/D cycles.

## 2. Materials and Methods

### 2.1. Organism and Culture Conditions

*Chromochloris zofingiensis* (SAG 211-14) was obtained from the Culture Collection of Algae at Goettingen University (SAG). The nutrient medium was mBG-11 [26]. Cultures were cultivated in glass columns (cylindrical, length: 63 cm or 31.5 cm; internal diameter: 3.5 cm) containing 200 or 500 mL, respectively. Illumination of 75 μmol photons m^−2^ s^−1^ (low light, LL) was provided by tubular cool daylight fluorescent lamps (Osram 18 W/765 or PHILIPS TLD 36 W/54) and illumination of 800 μmol photons m^−2^ s^−1^ (high light, HL) was provided by halogen floodlight lamp (Kengo 220–240 V, 1500 W). Light intensity was measured inside, at the center of an empty glass column, with a quantum meter (LI-250A Light Meter, LI-COR Inc., Lincoln, NE, USA). The columns were submerged in a temperature-controlled water bath kept at 25 °C. Mixing and CO_2_ supplementation were done by constant bubbling of air enriched with 2% CO_2_ (*v/v*). In case of cultivation under light/dark (L/D) regime (14 h of light and 10 h of dark), CO_2_ supply was stopped during the dark periods to prevent pH fluctuations.

#### 2.1.1. Batch Cultivation under Continuous Illumination

Exponentially grown cells (pre-grown under continuous LL illumination, in 200- or 500-mL glass columns for 2 days) were used as an inoculum. Inoculum was diluted with fresh medium to a cell density of approximately 2.5 × 10^6^ cell/mL and cultivated under the same conditions. Culture aliquots were sampled and stained with SYBR Green I to determine the nuclei number in cells.

#### 2.1.2. Batch Cultivation under L/D Illumination Regime

Cultures, raised as described above, were cultivated under LL intensity provided in L/D (14 h/10 h) cycle, until they reached the late stationary phase (14 days). These L/D adapted cultures were then diluted to a cell density of approximately 7 × 10^6^ cell/mL and were kept under the same conditions. Samples were taken for investigation during 14 days of experiment but only data from the first 6 days, which encapsulated most of the changes, are presented.

#### 2.1.3. Continuous Cultivation under L/D Illumination Regime

Cultures were grown under HL intensity, provided in an L/D (14 h/10 h) illumination regime. In order to get synchronization of growth and divisions with the L/D periods, cell concentration was adjusted daily to approximately 2 × 10^6^ cell/mL at the beginning of every illumination period. Under the L/D regime growth (G1) and divisions (DNArds) were spontaneously synchronized with the light and dark periods and in combination with the daily based dilution repeated themselves at every 24 h cultivation cycle. To establish the synchronization of the culture, the dilution was repeated daily for a minimum of 10 days and from the 11th day onward samples were withdrawn for analysis, and every 24 h cycle was considered as a repetition of the experiment.

### 2.2. Mean Light Intensity

The mean light intensity (I) was calculated as described in [27], according to the Beer–Lambert formula: I = (Ii − It)/ln(Ii/It). (Ii) represents illumination intensity measured at the surface of the culture vessel and (It) is the transmitted light intensity measured at the rear side of the culture vessel.

### 2.3. Cell Counting

The cell number was determined by Hemocytometer (Bright-Line, Hausser Scientific, Horsham, PA, USA). Specific growth rate (μ) was calculated based on the cell density.

### 2.4. Cell and Nuclei-Size Measurements

Average cell volume was calculated based on cell diameter measurements, which were taken manually using a microscope and ZEN 2012 software (Zeiss, Germany), considering the algal cell as a sphere. The diameters of nuclei with a clearly visible border were also manually measured. In cells containing more than 8 nuclei, not all nuclei were clearly visible and in 16, 32 and 64- PNC 12, 9 and 6 nuclei with a clear border were measured, respectively.

### 2.5. Nuclei Staining with SYBR Green I

SYBR Green I solution (Molecular Probes, Eugene, OR, USA, Ex/Em 497 nm/520 nm) was diluted 1:20 (*v/v*) in DMSO. For the staining of nuclei, 2.5 µL of the diluted SYBR Green I were added to 12.5 µL of culture samples. Samples were then incubated for 5 min at room temperature in the dark; fresh medium (100 µL) was added before microscope observations.

### 2.6. Microscopy and Image Processing

Images were acquired using Axio Imager A2 microscope coupled with an AxioCam and HXP-120 light source for fluorescence illumination (ZEISS, Oberkochen, Germany). Image acquisition and processing was conducted using ZEN 2012 (blue edition, version 1.1.2.0, ZEISS). The filter/analyzer cube sets used included DIC analyzer, Filter Set 38 (489038-9901-000), and Filter Set 16 (488016-9901-000) for the visualization of DIC, SYBR Green I and chloroplasts, respectively (ZEISS).

### 2.7. Statistical Analysis

Error bars, representing SE, were calculated based on a minimum of three replicates.

## 3. Results

### 3.1. Cell Morphology and Nuclear Status—Growth under Continuous LL Illumination

**Figure 1:**
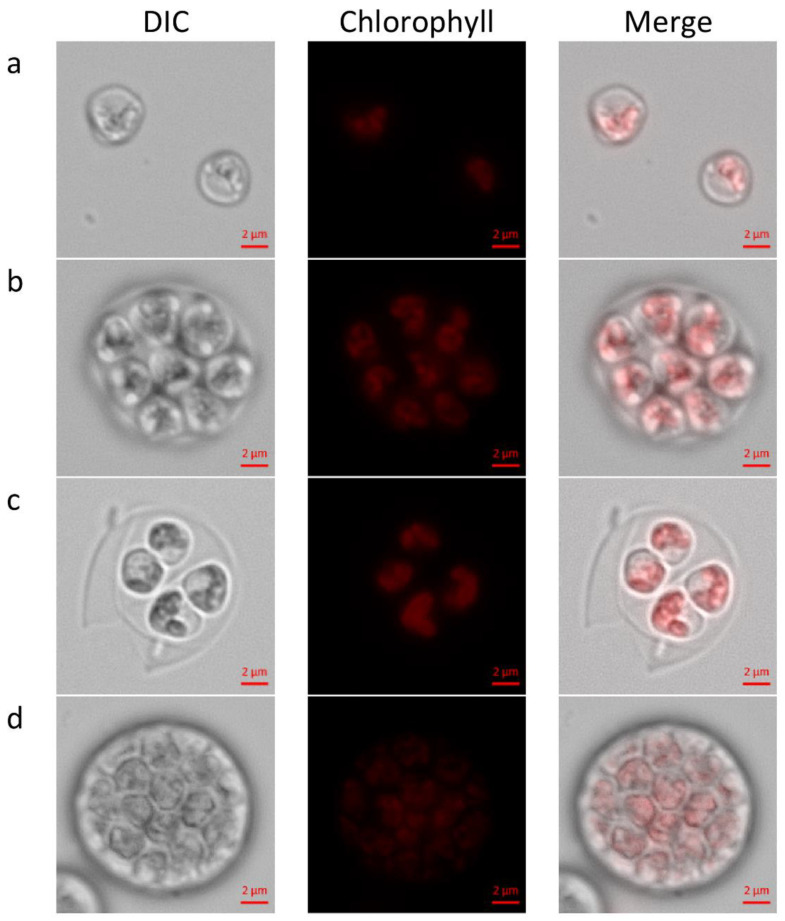
Previously reported *C. zofingiensis* morphologies. (**a**) autospores; (**b**) autosporangium, (**c**) autospores release, (**d**) forming autosporangium. DIC, differential interference contrast; Chlorophyll, chloroplast red auto-fluorescence signal. Scale bar = 2 µm.

**Figure 2:**
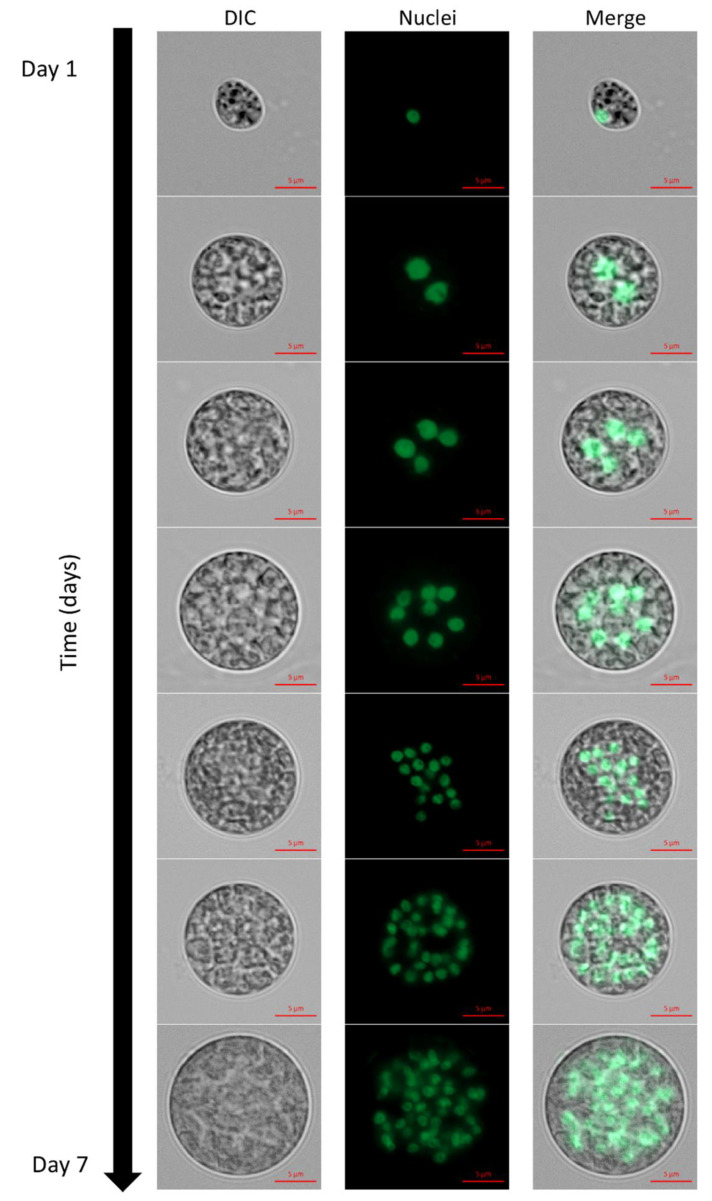
Cells with increasing size and increased numbers of nuclei were observed as the culture matured. During 6–7 days of cultivation under continuous illumination, mononuclear cells (**top**) to over 32 PNC (**bottom**) were observed. Nuclei fluorescence after staining with SYBR Green I; DIC, differential interference contrast. Scale bar = 5 µm.

The investigation began with the morphological characterization of *C. zofingiensis* cell types in batch cultures grown under continuous illumination of LL (Figure 1 and Figure 2). Under these conditions, number of previously reported [22,24] life-cycle stages and morphologies were observed, including: autospores, autosporangia and young autospore release (Figure 1). The autosporangia were observed with two autospores up to over 32 autospores (not shown). The chloroplasts’ structure and arrangement were consistent with previous reports [22,24] of parietal chloroplast/s in young autospores (Figure 1a,c). Chloroplasts that appeared to have a polygonal disk-like shape, as was described by Hindák (1982) [24], were observed in the forming autosporangia (Figure 1d).

Visualization of nuclei by fluorescent staining was conducted in order to characterize the nuclei dynamics accompanying the autosporangia formation. Under continuous illumination, cells with increasing number of nuclei appeared along with the progress in culture growth. Over 6 days of cultivation, mononuclear to over 32 and possibly even 64-PNC were observed (Figure 2). The exact number of nuclei in a large PNC is hard to determine due to overlaps between the nuclei, which are distributed throughout the cell volume. In some of the multinucleated cells, nuclei can be small (approximately 1.2–1.6 µm in diameter in 1, 16 and >16 nuclei containing cells, ) or enlarged (approximately 1.6–3 µm in diameter in 2–8 PNC, ). The arrangement of SYBR-stained nuclei in the center of PNC along with auto-fluorescence of the chloroplast can be seen in Figure S1.

### 3.2. Growth and Division Cycle of C. zofingiensis Batch Cultures under LL with L/D Illumination Regime

**Figure 3:**
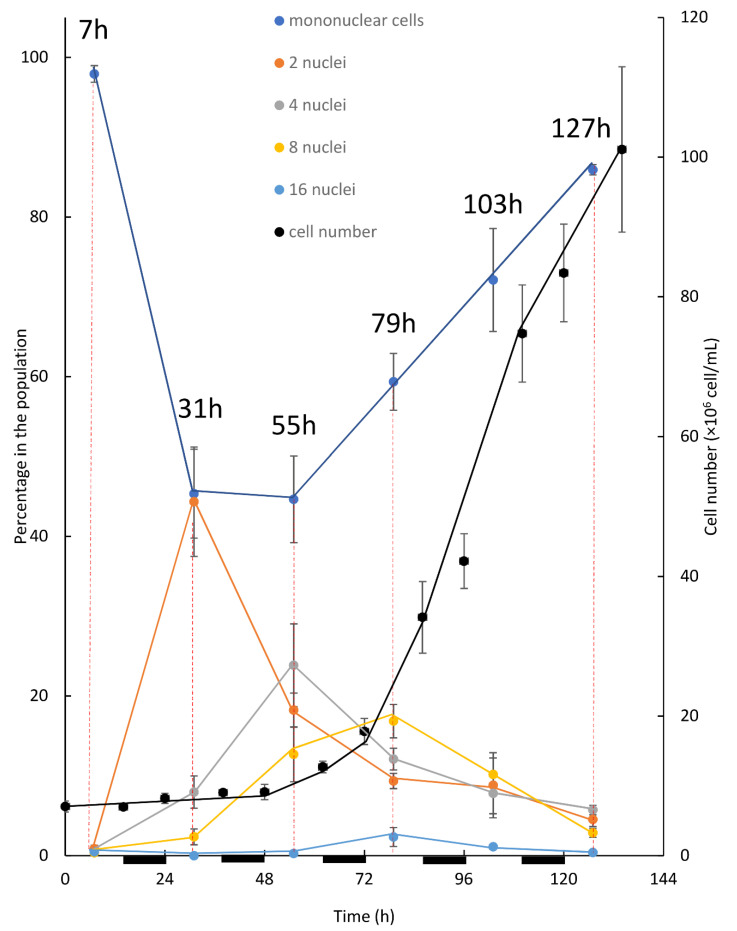
Cell number and percentage of mono/polynuclear cells subpopulations in a batch culture of *C. zofingiensis* under L/D illumination regime with LL. Dark periods are indicated by black lines under the X axis. To assess the distribution of PNC cells in the population samples were taken at the middle of each light period, and for each sample the nuclear status of 69–189 (120 ± 31, average ± SD) randomly observed cells was recorded. Each data point represents the average ± SE of three replicates.

To detect the native cell-cycle, we investigated *C. zofingiensis* grown under L/D (14 h/10 h) illumination regime in batch cultures (Figure 3). For this purpose, 2-week old, late stationary cultures grown under L/D illumination regime with LL were diluted to 7 × 10^6^ cells/mL. Cultures were monitored for 14 days and the percentage of the different mono/polynuclear cell populations relative to the entire cell population was recorded once a day (at the middle of the light period). The first 5–6 days, however, encompassed most of the changes and are presented in Figure 3. Shortly after inoculation, homogenous population of solitary mononuclear cells was evident and 97.9 ± 1% (average ± SE) of the population was composed of these types of cells (Figure 3, 7 h). The percentage of mononuclear cells in the population dropped 31 h after inoculation to 45.3 ± 5.6%; in parallel the percentage of binuclear cells increased and peaked at 44.3 ± 6.8% (Figure 3, 31 h). At the same time, the percentage of the 4 and 8-PNC increased from less than 1% to 8 ± 2% and 2.4 ± 1%, respectively, while 16-PNC were almost not detected. On the next day (Figure 3, 55 h), the percentage of the mononuclear cells (44.6 ± 5.4%) was similar to that observed in the day before; however, the percentage of binuclear cells dropped to 18.2 ± 2.1, while the percentage of 4, 8 and 16-PNC increased to 23.9 ± 5.2, 12.7 ± 3.4 and 0.3 ± 0.3%, respectively. In the next 24 h (Figure 3, 79 h), the percentage of mononuclear cells rose to 59.4 ± 3.6%, while the percentage of the binuclear cells decreased further to 9.4 ± 1%. At the same time, the percentage of 4-PNC also dropped to 12.1 ± 1.4%, while the percentage of 8 and 16-PNC increased to a peak of 16.9 ± 2.1% and 2.3 ± 1.2%, respectively. Interestingly, pronounced increase in the total cell number was only detected 48 h after cultivation and no autosporangia were visible earlier (not shown); cell number sharply increased only from time 72 h onwards (Figure 3, 72–110 h). A robust increase in population size occurred along with the decrease in the percentage of PNC subpopulations and the increase in the mononuclear subpopulation indicates cell divisions of the PNC. Interestingly, in this experimental setup, although the cultures were kept under L/D regime, no evidence indicated that cell divisions were synchronized with the dark periods (cell numbers were measured at the beginning and end of every dark period, Figure 3).

### 3.3. Growth and Division Cycle of C. zofingiensis Continuous Cultures under HL with L/D Illumination Regime

**Figure 4:**
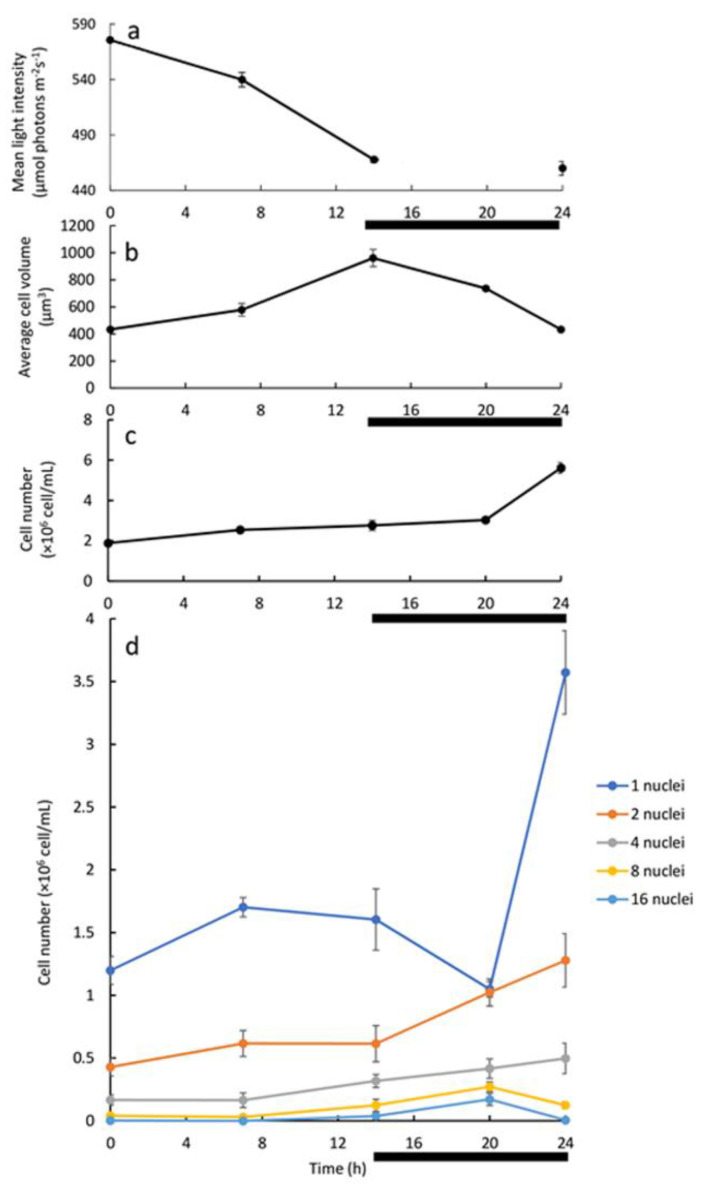
Continuous culture *of C. zofingiensis* under the 24 h L/D illumination regime under HL and with daily dilutions: (**a**) mean light intensity; (**b**) average cell volume; (**c**) total cell number; (**d**) the size of the mono/polynuclear cells subpopulations. The dark period is indicated by a black line under the X axis. Each data point represents the average ± SE of at least three 24 h cultivation cycles. To determine the average cell volume (**b**), around 100–300 random cells from each sample were measured. In (**d**), assessment of the mono/polynuclear cells subpopulations sizes is based on the examination of around 100–250 random cells from each sample. The cell number of every subpopulation was calculated from the percentage and the total cell number at every time point. 97.7%, 98.8%, 97.7%, and 96.8% of the observed cell subpopulations are included in the presentation at time-points 0/24 h, 7 h, 14 h, and 20 h, respectively. The neglected 1.2–3.2% populations are autosporangia and over 16-PNC. Each data point represents 3 repetitions derived from 3 cultivation cycles.

A different experimental setup of continuous growth mode was used, to further characterize the cell division pattern. Culture (~2 × 10^6^ cells/mL) was cultivated under L/D (14 h/10 h) illumination regime with HL, and daily diluted at the beginning of the light period to the initial cell concentration. After a minimum of 10 days of consecutive dilutions, measurements began (Figure 4). During the illumination period, the mean light intensity decreased from 575.6 ± 2.1 to 467.7 ± 2.6 μmol photons m^−2^ s^−1^ at time 0 and 14 h, respectively (Figure 4a, 0–14 h). During the same time-interval, the average cell volume increased from 434.7 ± 16.2 μm^3^ to 961.3 ± 63.2 μm^3^ (Figure 4b), and cell number increased by 50%, from 1.89 ± 0.13 × 10^6^ to 2.76 ± 0.25 × 10^6^ cells/mL (Figure 4c). During the dark period (14–24 h), the cell number increased further by 100% (5.62 ± 0.27 × 10^6^ cells/mL) while the average cell volume dropped back to its minimal value (Figure 4b,c). The major increase in cell number, from 3.03 ± 0.13 × 10^6^ to 5.62 ± 0.27 × 10^6^ cells/mL, along with the robust decrease in average cell volume (from 737 ± 16.5 to 434.7 ± 16.2 µm^3^), occurred during the last 4 h of the dark period (20 to 24 h), prior the onset of illumination (Figure 4b,c). The µ, which was calculated based on the change in cell number over a 24 h cycle (Figure 4c), was 0.04 h^−1^.

To better understand the underlying cell-cycle events under this growth system, nuclei were stained and visualized and the number of cells in the five major mono- and polynuclear subpopulations was assessed (Figure 4d). At the start of the cultivation cycle, along with the increase in total cell number (Figure 4c, 0–7 h), the mononuclear subpopulation cell number increased from 1.2 ± 0.11 to 1.7 ± 0.08 × 10^6^ cell/mL (Figure 4d, 0–7 h) and autosporangia were detected in the cultures (not shown); a slight increase in the number of binuclear cells was also observed. In the next 7 h of light period (Figure 4d, 7–14 h), the number of 4, 8 and 16 PNC increased slightly while mono- and binuclear cells population size was stable. Overall, during the light period changes in mono/polynuclear subpopulation size were relatively minor as compared to the changes during the dark period. During the dark period, the number of mononuclear cells dropped from 1.6 ± 0.24 × 10^6^ at 14 h to 1.05 ± 0.06 × 10^6^ cells/mL at 20 h, along with an increase in number of all other PNC (Figure 4d, 14–20 h). The sharp increase in total cell number (Figure 4c, 20–24 h) was coupled with a sharp increase in the number of the mononuclear cells (Figure 4d, 20–24 h), from 1.05 ± 0.06 × 10^6^ at 20 h to 3.57 ± 0.33 × 10^6^ cells/mL at 24 h. In parallel, the 8 and 16 PNC subpopulations size dropped from their peak values of 0.27 ± 0.04 × 10^6^ and 0.17 ± 0.05 × 10^6^ cells/mL at 20 h to 0.12 ± 0.03 × 10^6^ and 0.01 ± 0.01 × 10^6^ at 24 h, respectively (Figure 4d, 20–24 h). Based on our calculations, the decrease of approximately 0.15 × 10^6^ cells/mL in the eight PNC subpopulations and of approximately 0.16 × 10^6^ cells/mL in the 16 PNC subpopulation (Figure 4d, 20–24 h) is sufficient to support the increase of approximately 3.73 × 10^6^ cells/mL in total cell number along the 24 h cultivation cycle (Figure S1).

## 4. Discussion

The presented results are discussed in light of the published information [4,28] on microalgal cell-cycle in attempt to better characterize *C. zofingiensis* division pattern. Under continuous LL, we detected the previously reported [22,24,25,29] morphologies of newly hatched autospores and autosporangia (Figure 1a–d). We observed autosporangia, which released 2 to 32 and rarely even 64 autospores, as published earlier [23]. These observations indicate that *C. zofingiensis* can divide by either binary or multiple fission division patterns. As discussed in Bišová and Zachleder (2014) [4], the number of daughter cells is correlated with the culture growth rate in microalgae that can perform both binary and multiple fission. Higher growth rates are associated with bigger population of large autosporangia that can divide into several (more than two) autospores. We suggest that under the conditions of batch culture and LL, at the beginning of cultivation, the growth of *C. zofingiensis* was unlimited by the conditions, and the cultures attained high growth rate and thus cells divided by multiple fission. At the plateau stage, as the culture become dense and turbidity of the culture is relatively high, light became limiting and growth rate declined so that binary fission was executed. Chloroplast dynamics during cell-cycle was not recorded in the current study. Nevertheless, the previously reported observations of parietal chloroplast/s in young autospores (Figure 1a–c) and polygonal disk-like shaped chloroplasts in the forming autosporangia (Figure 1d) were detected and verified [22,25,29].

The nuclei visualization protocol that we developed in this study enabled, for the first time, the detection of mono and PNC along the cultivation period and the assessment of nuclei sizes. Under the examined conditions, a single cell-cycle of *C. zofingiensis* can spread over a period of several days; during this period cells’ size enlarged (Figure 2), indicating the progress in G1 phase. We suggest that, with every approximate doubling of size, CP is attained. According to our observations, up to five or six CPs can be attained during a single cell-cycle of *C. zofingiensis*, and subsequently facilitate the formation of up to 32–64 autospores. As mentioned previously [4], every CP attained should be followed by DNArds. The detected multinucleated stages and differences in the sizes of nuclei among the PNC indicate that DNArds are being executed in the cells in a consecutive pattern. We suggest that the observed large nuclei (approximately 1.6–3 µm in diameter, Figure 2, 2–8 PNC) represent cells containing two copies of DNA during and after S phase and before M phase, and the observed small nuclei (approximately 1.2–1.6 µm in diameter, Figure 2, 1, 16 and >16 nuclei containing cells) represent cells before S phase or after M phase is completed. In principle, the cell undergoes multiple rounds of S phase before it divides and autosporangia are mainly visible in 8 and 16 PNC. Such consecutive division pattern described herein for *C. zofingiensis* is similar to its close relative *Scenedesmus,* but contradicts previous suggestions [19] comparing it to the model green alga *C. reinhardtii.*

The results discussed above indicate that *C. zofingiensis* can divide by binary or multiple fission in a consecutive pattern. Continuous illumination, however, is unnatural and therefore may not support reliable characterization of the division pattern. Moreover, continuous illumination is of a lesser relevance to outdoor mass culture applications. More relevant to natural behavior and realistic application is the investigation of *C. zofingiensis* cell-cycle under alternating L/D regime.

**Figure 5:**
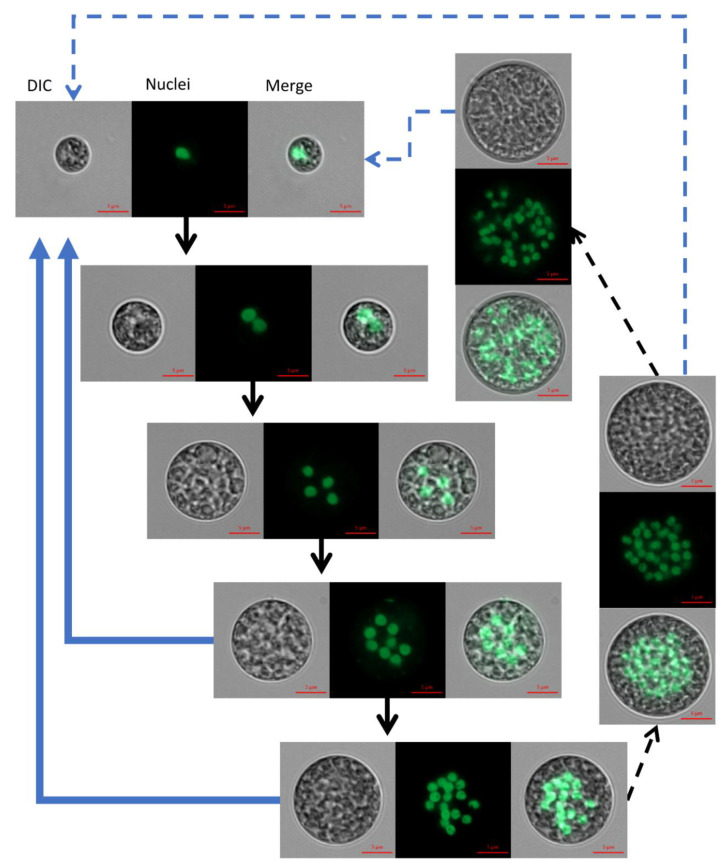
A model representing the cell-cycle of *C. zofingiensis*. As the cells progress through the cell-cycle, grow, commit to divide, execute DNA replications and nuclear divisions, they create the PNC sub-populations (thick black arrows). Cytokinesis is prevalent among the 8 and 16-PNC (thick blue arrows). As a minor phenomenon, cells can grow and progress in the cycle to become 32 and possibly 64-PNC (thin dashed black arrows) and these cells can also divide (thin dashed blue arrows). Nuclei are stained with SYBR Green I, DIC-differential interference contrast. Scale bar = 5 µm.

The first experimental setup for investigating the pattern of division under an alternating L/D regime was performed in batch mode. In this setup (Figure 3), after inoculation the dominant subpopulation of solitary mononuclear cells initiated their first growth phase (G1). Throughout the G1 phase, as cells doubled in volume (not shown), the first CP was attained. Consequently, DNArds was initiated and included execution of S, G2, and M phases, giving a rise to the binuclear cells. Evidently, the peak of binuclear cells subpopulation (observed at 31 h), along with the reduction in the mononuclear cell subpopulation, indicate the transition of cells from one subpopulation to the other. Cell number did not increase at this stage, indicating that cytokinesis was not yet conducted. It appears that instead, a second G1 phase was started with the attainment of the first CP, leading to a second doubling in cell volume and the attainment of a second CP. The second CP was, like the first one, accompanied by the initiation of a third G1 phase. Binuclear cells, which doubled their volume in their second G1 phase, started executing a second DNArds, giving a rise to the four-nuclei PNC, and in most of the cells cytokinesis was not yet conducted. Supporting this assumption is the reduction in the percentage of binuclear cells, along with the increase—to a peak value—of the 4-nuclei PNC, indicating the flow from one sub-population to the other. In the same manner, it can be suggested that the 4-nuclei PNC, after attaining the third CP, grew to around 8 times the volume of an autospore and executed a third DNArds to give rise to the 8-nuclei PNC. Again, supporting this assumption is the drop in the percentage of the 4-nuclei PNC subpopulation to a peak value, indicating the flow from one population to the other. The results suggest that some of the cells progressed more in the cell-cycle, and therefore conducted three G1 phases and attained three CPs; these cells were able to grow and subsequently committed to divide once again in order to give rise to the 16-nuclei PNC. The 16-nuclei PNC, in turn, produced 16 autospores via large-volume (16 times the volume of an autospore) autosporangia, which were visible under the microscope (not shown). The 16-PNC subpopulation started to grow at around 55 h after cultivation and expanded to a peak level at 79 h. According to the suggested explanation, the 16-PNC detected at 55 h are at the cutting edge of progress in cell-cycle and are followed in level of progression by the 16-PNC detected at 79 h. As indicated by an increase in cell number, the assessment of total population growth relative to changes in the size of the 5 subpopulations composing it, revealed a clear pattern. The increase in cell number and the increase in the mononuclear subpopulation temporally correlated with the expansion of 8 and 16-PNC subpopulations. This pattern continued and became more profound as the 8 and 16-PNC subpopulations decreased in size, suggesting a flow from these subpopulations to the mononuclear subpopulation via cell division. Thus, a cell from the 8- nuclei PNC subpopulation can have two possible fates. If culture conditions permit growth at a sufficient level during the third G1 phase [4,11], a fourth CP will be attained and a fourth G1 phase will be initiated (usually without attainment of additional CP). Subsequently, the 8-PNC subpopulation would be converted to the 16-PNC subpopulation (Figure 5). Alternatively, if growth is not permitted at the sufficient level [4,11], the fourth CP will not be attained and the third DNArds will be terminated with C phases (as demonstrated in the model, Figure 5).

As part of this experimental setup, cultures were kept under alternating light and dark periods and cell numbers were measured at the beginning and at the end of the dark period, such that cell division in the dark could be assessed (Figure 3). Even though the phenomenon under which algal cells dividing by multiple fission synchronize growth and divisions with the available illumination regime is well known [4,11], such synchronization was not observed. It can be suggested that the batch cultivation included a constant change in culture conditions, as throughout the cultivation cells presumably increased in size and/or total cell density increased. As the cultures were kept under LL, it is likely that such changes could affect the available light per cell profoundly. In addition, as the batch mode does not include refreshment of the media, it is possible that the depletion of nutrients and the accumulation of waste products can also contribute to the instability of culture conditions overtime. Under this constant change, as every light and every dark period are different, synchronization of cell division with the light regime was not established.

**Figure 6:**
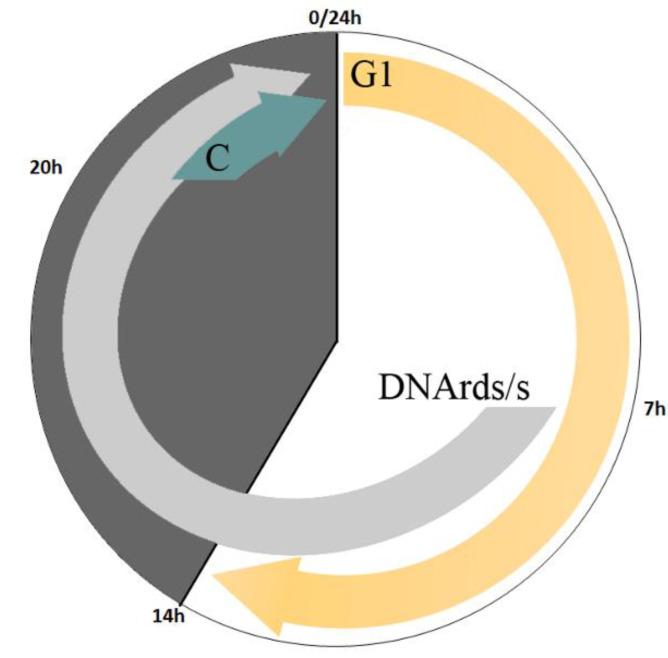
A model presenting cell-cycle events during the 24 h cultivation cycle in the continuous culture under D/L regime. During the 14 h light period, cells conduct G1 phase/s (yellow arrow). From the 7 h of light period and up to the end of the dark period, cells which grew sufficiently to attain CP/s will execute DNArds/s (gray arrow); at this time interval nuclear divisions are evident. From the 6 h of dark period cells, that reached a level permitting division into 8 or 16 autospores, will terminate progression in the cell-cycle and execute the C phase (blueish arrow).

Analysis of the batch culture grown under L/D illumination regime may suggest association between the development and division of 8 and 16-PNC with the release of autospores. The batch cultivation, however, facilitates constant changes in conditions as stated above. To overcome the limitations of the two above-mentioned experimental setups (batch under continuous LL and batch under L/D with LL), a 24 h cultivation cycle with alternating 14 h-light and 10 h-dark and dilution at the end of every dark period was established. Under this continuous experimental setup, cell density was kept low during experiment, with a maximum density of 5.62 ± 0.27 × 10^6^ cells/mL (Figure 4c) under HL, ensuring that considerable light limitation would not occur. Indeed, the change in the mean light intensity was not significant; it decreased from 575.6 ± 2.1 to 467.7 ± 2.6 μmol photons m^−2^ s^−1^ during the 14 h light period (Figure 4a, 0–14 h). The daily dilution, replacing around 65% of the culture media every 24 h, ensured that all nutrients are available, and no waste builds up. It can be argued that under this experimental setup there is no abiotic growth limitation. Indeed, the µ, which was calculated from the increase in cell number during the 24 h L/D cycle (Figure 4c), of 0.04 h^−1^, is the highest µ ever reported for the photoheterotrophic cultivation of *C. zofingiensis* [14,30]. Under this setup *C. zofingiensis* cells spontaneously synchronized their growth with the light period and cell divisions with the dark period, as depicted in our schematic model (Figure 6). Increase in turbidity and average cell volume without a major increase in cell numbers along the light period indicate that the cells are mostly growing during the light period (Figure 4). We thus suggest that a G1 phase is being conducted. However, in the dark period, an increase in cell number coupled with a decrease in the average cell volume, particularly during the last 4 h of the dark period was observed, indicate that cells terminate cell-cycle and divide in the dark period. Nuclei visualization was conducted in order to capture the pattern of cell-cycle events under this experimental setup. As the G1 progressed, CP was attained with every approximate doubling of size, but DNArds were executed mainly during the dark period, though a minor population of cells executed DNArds after 7 h in the light. In the darkness, the mononuclear cells subpopulation decreased with a simultaneous increase in the PNC subpopulations, indicating a flow from mono to PNC. During the last 4 h of dark, a sharp increase in total cell number, coupled with a sharp increase in the number of the mononuclear cells, and a decrease in the number of the 8 and 16 PNC, indicate that these cells divided and released autospores. Therefore, it seems that under this experimental setup, the major PNC subpopulations that divide are the 8 and 16-PNC (Figure 4, Figure 5 and Figure S2).

As results indicate that large cells, which makeup a small portion of the total population, are mostly dividing at the end of the dark period, and as our measurements show that volume increase of 8–16 times is not feasible during one light period (Figure 4b), the average cell probably undergoes several L/D cycles to complete one cell-cycle event. Under the examined conditions of daily dilution and simultaneous exposure to HL, it is possible that excessive light stress is imposed to the cells and therefore cell volume does not increase enough during the light period to allow faster growth. As a matter of fact, the small increase in cell number in each L/D cycle (three-fold increase) with no peak of 8/16 PNC in the light period may indicate a growth inhibition/HL acclimation at the onset of the daily dilution. To summarize, the combined measurements of cell number, cell size, and number of nuclei per cell under different cultivation setups helped us to elaborate the pattern by which *C. zofingiensis* grow and divide. Since the PNC were observed under both, high and low light conditions, and under both batch and continuous culture, we conclude that light limitations or change in extra cellular conditions (such as nutrient limitation or change in pH) do not bias our findings, and the cell cycle pattern describe herein is an intrinsic property of this alga. Further studies are needed to explore and understand the underlying mechanism of the consecutive cell cycle division in *C. zofingiensis.*

## 5. Conclusions

*C. zofingiensis* (Chlorophyceae) divides by consecutive multiple fission cell-cycle under batch and continuous cultivation.

SYBR green—applied in DMSO solution—allowed us to visualize polynuclear cells of *C. zofingiensis.* We thus concluded that this alga divides by a consecutive pattern of multiple fission. In high-light continuous culture or low-light batch culture, *C. zofingiensis* cell-cycle was completed within several light-dark (L/D) cycles (14 h/10 h), however cell divisions were synchronized with the dark periods only in the high-light continuous culture. In both modes of cultivation, daughter cell release was mainly facilitated by division of 8 and 16-polynuclear cells.

## Data Availability

Data sharing not applicable.

## Supplementary Materials

The following are available online at https://www.mdpi.com/2079-7737/10/2/157/s1, Figure S1: The arrangement of SYBR green stained nuclei in the center of PNC along with auto-fluorescence of the chloroplasts. Chlorophyll auto-fluorescence (red) and SYBR fluorescence are imaged using excitation wavelength of 493 nm and emission of 517 nm. Upper panel, differential interference contrast; lower panel, fluorescence. Scale bar = 5 µm. Figure S2: The 8 and 16-PNC that disappear from the population at the end of the dark period (Figure 4d, 20–24 h) may account for the increase in total cell number along the 24 h cycle. * The decrease in the number of 8 and 16 PNC in the last 4 h of the dark period is considered as if they were developed into autospores-releasing autosporangia.
