# Supplementary material for: Chromochloris zofingiensis (Chlorophyceae) Divides by Consecutive Multiple Fission Cell-Cycle under Batch and Continuous Cultivation
Source: Biology (Basel). 2021 Feb 16;10(2):157. doi: 10.3390/biology10020157 (PMC7920477; doi:10.3390/biology10020157)
Supplement: Supplementary file 1 [file biology-10-00157-s001.pdf]

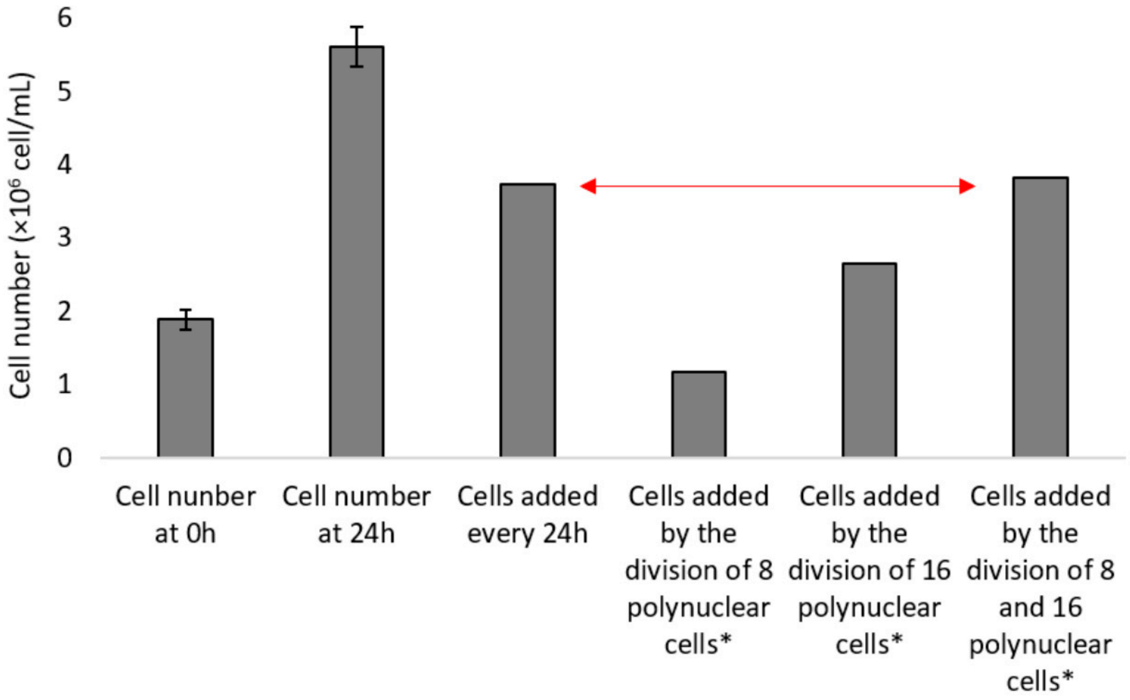

Figure S1. The arrangement of SYBR green stained nuclei in the center of PNCs along with auto-fluorescence of the chloroplasts. Chlorophyll auto-fluorescence (red) and SYBR fluorescence are imaged using excitation wavelength of 493 nm and emission of 517 nm. Upper panel, differential interference contrast; lower panel, fluorescence. Scale bar = 5 μm.

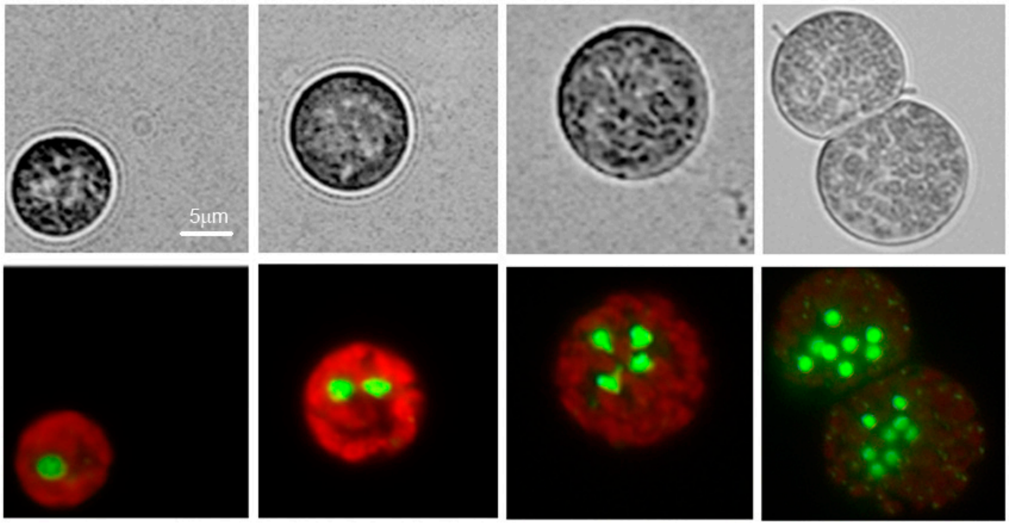

Figure S2. The 8 and 16-PNC that disappear from the population at the end of the dark period (Fig. 4, d, 20-24h) may account for the increase in total cell number along the 24h cycle. *The decrease in the number of 8 and 16 PNC in the last 4h of the dark period is considered as if they were developed into autospores-releasing autosporangia.
